# The role of spermatozoa-zona pellucida interaction in selecting fertilization-competent spermatozoa in humans

**DOI:** 10.3389/fendo.2023.1135973

**Published:** 2023-03-20

**Authors:** Erica T. Y. Leung, Brayden K. M. Lee, Cheuk-Lun Lee, Xinyi Tian, Kevin K. W. Lam, Raymond H. W. Li, Ernest H. Y. Ng, William S. B. Yeung, Jian-Ping Ou, Philip C. N. Chiu

**Affiliations:** ^1^ Department of Obstetrics and Gynaecology, Li Ka Shing Faculty of Medicine, The University of Hong Kong, Hong Kong, Hong Kong SAR, China; ^2^ Shenzhen Key Laboratory of Fertility Regulation, The University of Hong Kong – Shenzhen Hospital, Shenzhen, China; ^3^ Center for Reproductive Medicine, The Third Affiliated Hospital, Sun Yat-sen University, Guangzhou, China

**Keywords:** zona pellucida, human spermatozoa, sperm selection, DNA integrity, methylation, protamination, HSPA2, SPACA3

## Abstract

Human fertilization begins when a capacitated spermatozoon binds to the zona pellucida (ZP) surrounding a mature oocyte. Defective spermatozoa-ZP interaction contributes to male infertility and is a leading cause of reduced fertilization rates in assisted reproduction treatments (ARTs). Human ejaculate contains millions of spermatozoa with varying degrees of fertilization potential and genetic quality, of which only thousands of motile spermatozoa can bind to the ZP at the fertilization site. This observation suggests that human ZP selectively interacts with competitively superior spermatozoa characterized by high fertilizing capability and genetic integrity. However, direct evidence for ZP-mediated sperm selection process is lacking. This study aims to demonstrate that spermatozoa-ZP interaction represents a crucial step in selecting fertilization-competent spermatozoa in humans. ZP-bound and unbound spermatozoa were respectively collected by a spermatozoa-ZP coincubation assay. The time-course data demonstrated that ZP interacted with a small proportion of motile spermatozoa. Heat shock 70 kDa protein 2 (HSPA2) and sperm acrosome associated 3 (SPACA 3) are two protein markers associated with the sperm ZP-binding ability. Immunofluorescent staining indicated that the ZP-bound spermatozoa had significantly higher expression levels of HSPA2 and SPACA3 than the unbound spermatozoa. ZP-bound spermatozoa had a significantly higher level of normal morphology, DNA integrity, chromatin integrity, protamination and global methylation when compared to the unbound spermatozoa. The results validated the possibility of applying spermatozoa-ZP interaction to select fertilization-competent spermatozoa in ART. This highly selective interaction might also provide diagnostic information regarding the fertilization potential and genetic qualities of spermatozoa independent of those derived from the standard semen analysis.

## 1 Introduction

Infertility is a public health concern affecting approximately 15% of reproductive-aged couples worldwide (1). Assisted reproduction technologies (ARTs) including *in vitro* fertilization (IVF), intrauterine insemination (IUI), intracytoplasmic sperm injection (ICSI) are provided to infertile couples who wish to conceive. Despite major technological advancements, the live birth rate following ART is only about 30% (2).

Sperm quality is a major factor contributing to fertilization success following ARTs (3, 4). During migration through the female reproductive tract, only the fertilization-competent spermatozoa survive the natural selection mechanisms involving anatomical, biochemical, and physiological barriers in a highly specialized microenvironment (5). In ART, the motile and morphologically normal spermatozoa are routinely isolated by either the swim-up or density gradient centrifugation (DGC) according to their motility and density, respectively for subsequent procedures. However, these two surrogate markers lack the discriminatory power to select high-quality spermatozoa according to their fertilization potential and genetic quality (5–10).

Advanced sperm selection methods have been developed to enrich high-quality spermatozoa aiming to improve the fertilization and clinical outcomes (11–14). Two approaches are being used to develop sperm selection methods. One approach is to focus on the intrinsic properties of spermatozoa, such as surface charges and apoptotic status of spermatozoa (15, 16). Another approach is to replicate the natural selection mechanisms such as thermotactic and chemotactic responses in a controlled environment to isolate fertilization-competent spermatozoa in a manner similar to those observed in the female reproductive tract (5, 17, 18). However, contradicting results have been reported on the clinical significance of these methods on ART outcomes (5). In addition, many of these new techniques are small-scaled, which can only process a small volume of sample per run (5). These findings highlight the need for new approaches to sperm selection in ART.

In the fallopian tubes, the capacitated spermatozoa bind to the zona pellucida (ZP) surrounding the released oocyte and undergo acrosome reaction which facilitates the subsequent penetration process for gamete fusion. Defective sperm-ZP binding (DSZPB) is a leading cause of male-factor infertility and fertilization failure in IVF (19–22). Spermatozoa-ZP binding is a highly species specific event that occurs when the specific ligands on the ZP are recognized by the protein receptors located on the capacitated spermatozoa (23). The receptor(s) are multimeric protein complex(es) assembled during capacitation (24). Fertile male ejaculate contains millions of spermatozoa, of which only 14% of the motile spermatozoa are capable of binding to the ZP (25) and only 48% of the ZP-bound spermatozoa can then undergo ZP-induced acrosome reaction (26). These observations suggest that human ZP selectively interacts with high-quality spermatozoa possessing superior genetic integrity and fertilizing capability. However, direct evidence for the existence of this ZP-mediated sperm selection process in humans is lacking. Therefore, the objective of this study is to validate the functional role of human ZP in selecting fertilization-competent spermatozoa.

## 2 Materials and methods

### 2.1 Semen and oocyte collection

The research protocol of this study was approved by the Institutional Review Board of the University of Hong Kong/Hospital Authority Hong Kong West Cluster. Semen samples were collected from men attending the Family Planning Association of Hong Kong for premarital checkup after obtaining informed written consent. The residual portion after the routine test was collected for research use. Normal semen samples were selected for research use according to the World Health Organization (WHO) criteria, fifth edition (27): total volume > 1.5 mL, total motility > 40%, progressive motility > 32%, total sperm number > 39 × 10^6^ per ejaculate, concentration > 15 × 10^6^/mL, and viability > 58%, normal morphology > 4%. Direct swim-up method was used to isolate motile and viable spermatozoa from the seminal plasma. In this method, 1.0 mL of thoroughly mixed semen was overlaid by 1.5 mL of Earle Balanced Salt Solution (EBSS; Flow Laboratories, Irvine, United Kingdom) supplemented with 0.3% bovine serum albumin (BSA), 0.3 mmol/l sodium pyruvate, 0.16 mmol/L penicillin G, 0.05 mmol/l streptomycin sulfate, and 14 mmol/L sodium bicarbonate (all from Sigma, MO, USA) (EBSS/0.3% BSA). The tube was then placed at a 45° angle and incubated at 37°C with 5% CO_2_ for one hour to allow motile spermatozoa to migrate from the seminal plasma into the overlaying medium. 1 mL of the top layer of the medium was collected, transferred into a sterile 15 mL centrifuge tube, and centrifuged at 500g for 5 min twice. The washed pellet was then resuspended with EBSS/0.3% BSA and diluted to the appropriate concentrations (1 × 10^6^ or 2 × 10^6^ spermatozoa/ml). All processed spermatozoa were incubated in EBSS supplemented with 3% BSA (EBSS/3%BSA) to induce capacitation for the spermatozoa-ZP coincubation assays (28).

Oocytes were collected from infertile women attending IVF treatment at the Queen Mary Hospital, Hong Kong. Immature oocytes (germinal vesicle/metaphase I oocytes) or mature metaphase II oocytes that failed to achieve fertilization following conventional insemination were donated from patients with informed consent and stored in high-salt oocyte storage buffer containing 1.5 M MgCl_2_, 0.1% polyvinyl pyrrolidone (PVP) and 40 mM HEPES with pH 7.2. Morphologically abnormal oocytes were discarded.

### 2.2 Evaluation of sperm motility

Sperm motility was assessed by the computer-assisted sperm analysis (CASA) system (CEROS system, Hamilton Thorne, MA, USA). For each measurement, 10 μL of sperm sample was transferred on a glass slide (Hamilton Thorne, USA) specifically made for the CASA system equipped with a warmed stage at 37°C. A minimum of 200 spermatozoa per sample in randomly selected fields were examined to analyze the following sperm motion parameters: 1) Progressive motility (%), 2) average path velocity (VAP, μm/s), 3) straight line velocity (VSL; μm/s), 4) curvilinear velocity (VCL, μm/s), 5) lateral amplitude (μm), 6) beat frequency (Hz), 7) straightness (%), 8) linearity (%), 9) elongation of sperm head (%), and 10) area (μm).

### 2.3 Evaluation of sperm viability

Sperm viability was assessed by the trypan blue exclusion assay. Spermatozoa were thoroughly mixed with an equal volume of trypan blue dye (1:1 ratio) on a sterile glass slide and kept at 37°C for 5 min prior to examination. The mixture was then evaluated under a light microscope with a magnification of 400x. Viable spermatozoa appeared transparent whilst non-viable spermatozoa with disrupted plasma membranes were stained blue. A minimum of 200 spermatozoa per sample in randomly selected fields were quantified to determine the overall viability.

### 2.4 ZP-bound spermatozoa collection

ZP-bound spermatozoa were collected by a modified spermatozoa-ZP coincubation assay (25). In brief, 4 human oocytes were transferred into a 30 μL droplet of EBSS/3% BSA containing 2×10^6^ spermatozoa covered with mineral oil for coincubation at 37°C in 5% CO_2_ for 30 min unless stated otherwise. After incubation, the oocytes were successively washed with 3 droplets of EBSS/no BSA to dislodge loosely bound spermatozoa. The ZP-bound spermatozoa were then removed from the surface of the oocytes through vigorous aspiration using a glass pipette in a confined area containing 10 μL of EBSS/no BSA on a sterile glass slide for further analysis.

### 2.5 ZP-unbound spermatozoa collection

ZP-unbound spermatozoa were collected by a modified continuous spermatozoa-ZP coincubation assay (25). In this method, 4 human oocytes were transferred into a 20 μL droplet of EBSS/3%BSA containing 1×10^6^ spermatozoa for a 6-h incubation at 37°C in 5% CO_2_. The following procedures were performed at intervals of 2, 4 and 6 h. The oocytes were successively washed with 3 droplets of EBSS/no BSA to dislodge any loosely bound spermatozoa and transferred into a fresh droplet of EBSS/no BSA for observation under a light microscope. Only spermatozoa tightly bound to the ZP with their sperm heads were quantified whilst those bound to the ZP with their tails, or any other regions were omitted. The ZP-bound spermatozoa were then removed from the ZP as described above and the oocytes were transferred back into the original sperm droplet for further coincubation. All spermatozoa remaining in the sperm droplet after 6 h of coincubation were considered as unbound spermatozoa, which were subsequently collected for further analysis.

### 2.6 Evaluation of the acrosomal status of spermatozoa

The acrosomal status of spermatozoa was evaluated by the fluorescein isothiocyanate conjugated *Pisum sativum* (FITC-PSA) (Sigma) staining. 20 μL of ZP-bound and unbound spermatozoa were allowed to air-dry in a small area on a sterile glass slide at 37°C. The slide was then washed with distilled water thrice. The spermatozoa were incubated with 50 μL of FITC-PSA (20 μg/mL) for 1 h at 37°C in the dark. The slides were rinsed with distilled water twice and mounted with DAKO mounting solution (Dako, CA, USA). The stained spermatozoa were observed under a fluorescence microscope (Zeiss, Oberkochen, Germany) at a magnification of 600x using excitation/emission wavelengths of 495 nm/515 nm. Acrosome-intact spermatozoa were stained with bright green, fluorescent signals in no less than half of the acrosomal regions whilst acrosome-reacted spermatozoa only had fluorescent signals at the equatorial region or completely lacked fluorescent signals in the acrosomal region. A minimum of 100 spermatozoa/sample was quantified to determine the acrosome reaction rates.

### 2.7 Immunodetection of protein expressions on spermatozoa

Capacitated sperm suspensions were fixed with 0.4% paraformaldehyde for 10 min and washed with EBSS/no BSA twice. The fixed spermatozoa were individually incubated with the primary antibodies against heat shock 70 kDa protein 2 (HSPA2) (Sigma) or sperm acrosome associated 3 (SPACA 3) (Abcam, Cambridge, UK) diluted at 1:100 for 1 h at 37°C in 5% CO_2_. The samples were then centrifuged twice at 500g for 5 min and incubated with Alexa Fluro 488 conjugated goat anti-rabbit IgG for one hour at 37°C in 5% CO_2_. (1:1000; Invitrogen, MA, USA). The samples were centrifuged twice at 500g for 5 min and resuspended into EBSS/ no BSA for flow consistency (Beckman Coulter, CA, USA). Data were analyzed using FlowJo v10.0.7 software (Copyright Tree Star, Inc. Stanford Jr. University).

ZP-bound and unbound spermatozoa were allowed to air-dry in a small area on a sterile glass slide at 37°C. The slides were gently rinsed with distilled water thrice. The samples were individually incubated with primary antibodies against HSPA2 (Sigma) or SPACA3 (Abcam) diluted at 1:100 overnight at 4°C. The slides were gently rinsed with distilled water twice. The samples were then incubated with Alexa Fluro 555 conjugated goat anti-rabbit IgG for one hour at 37°C in 5% CO_2_. (1:1000; Invitrogen), rinsed gently with distilled water twice and immediately mounted with fluorescent mounting medium (Dako). The stained spermatozoa were observed under a fluorescence microscope (Nikon Eclipse Ti, Tokyo, Japan) at a magnification of 600x or a Carl Zeiss LSM 880 with Ariyscan 2 at a magnification of 630x (Carl Zeiss, NY, USA) using excitation/emission wavelengths of 555 nm/580 nm. A minimum of 100 spermatozoa/sample with positive signals across their head regions was quantified to determine the expression patterns. The fluorescence intensities were evaluated using Image J analysis software (Version 1.48 v; NIH, USA)

### 2.8 Evaluation of sperm morphology

ZP-bound and unbound spermatozoa were smeared on a glass slide and allowed to air-dry at 37°C. The slides were then gently rinsed with distilled water once. The samples were fixed with the fixative solution (methanol-based solution) for 15 s and stained with the staining solution 1 (buffered solution of Eosin Y) for 10 s followed by the staining solution 2 (buffered solution of thiazine dyes) for 10 s. The excess solution was allowed to drip off the slides between steps. The samples were gently rinsed with distilled water once and allowed to air-dry. Spermatozoa were assessed manually under oil immersion using a light microscope (Zeiss, Gottingen, Germany) at a magnification of 1000x. A minimum of 100 spermatozoa/sample was quantified according to the WHO strict criteria (27) to determine the percentage of normal morphology.

### 2.9 Evaluation of DNA fragmentation rates by terminal deoxynucleotidyl transferase dUTP nick end labelling

ZP-bound and unbound spermatozoa were allowed to air-dry in a small area on a sterile glass slide at 37°C. The slides were then gently rinsed with distilled water thrice. The washed spermatozoa were fixed with 2% paraformaldehyde for 1 h at room temperature and gently rinsed with PBS twice. After fixation, the spermatozoa were permeabilized with freshly prepared 0.1% Triton X-100 in sodium citrate for 2 min at 4°C and rinsed with PBS twice. The permeabilized spermatozoa were incubated in 50 μL of reaction mixture containing terminal deoxynucleotidyl transferase (TdT) and fluorescein- dUTP (*In Situ* cell death detection kit, fluorescein, Sigma-Aldrich, MA, USA) in a 1:9 ratio for 1 hour at 37°C in the dark. The slide was then rinsed with PBS twice and mounted with fluorescent mounting medium (Dako). The TUNEL-positive spermatozoa were observed under a fluorescence microscope (Nikon Eclipse Ti, Tokyo, Japan) at a magnification of 600x using excitation/emission wavelengths of 488nm/530nm. A minimum of 100 spermatozoa/sample was quantified to determine the percentage of DNA fragmentation rates [(number of TUNEL-positive spermatozoa with bright, green fluorescence over the head regions/total number of spermatozoa) x 100%].

### 2.10 Evaluation of DNA damages by comet assay

ZP-bound and unbound spermatozoa were mixed with the molten low-melting point agarose (Trevigen, MD, USA) at 1:5 ratio (V/V) on a Comet slide (Trevigen). The mixture was allowed to solidify at 4°C in the dark for 10 min. The slides were then immersed in cold lysis buffer (Trevigen) at 4°C for 30 min. After removing the excess buffer, the slides were incubated in freshly prepared alkaline solution (pH~13) for 30 min. The slides were then transferred into a horizontal electrophoresis tank containing freshly prepared alkaline buffer for electrophoresis at 21V for 30 min. The slides were washed in distilled water twice for 10 min followed by 70% ethanol for 5 min. The samples were allowed to air-dry at 37°C. The dried samples were then incubated with SYBR Green I (Molecular Probes, OR, USA) at room temperature in the dark for 5 min. After removing the excess staining solution, the COMET-positive spermatozoa were observed under a fluorescence microscope (Nikon Eclipse Ti, Tokyo, Japan) at a magnification of 600x using excitation/emission wavelengths of 488nm/530nm. The extent of DNA damage in a minimum of 100 spermatozoa/sample was determined by measuring the percentage of tail DNA ((tail intensity/total intensity) x 100%) and the tail moment (%DNA in the comet tail x tail length) using the COMET assay II software (Perceptive Instruments, Haverhill, UK).

### 2.11 Evaluation of chromatin integrity by acridine orange staining

Air-dried and washed ZP-bound and unbound spermatozoa in a small area on a sterile glass slide as described above were fixed with Carnoy’s solution (acetic acid-methanol in a 3:1 ratio) overnight at 4°C. The slides were gently rinsed with PBS twice and allowed to air-dry prior to the staining procedure. The AO staining solution was prepared daily by adding 1% AO stock solution (Thermofisher, MA, USA) to a mixture of 0.1M citric acid and 0.3M Na_2_HPO_4_, pH 2.5. The spermatozoa were incubated with the staining solution for 5 min at 37°C in the dark and gently rinsed with distilled water twice. The stained spermatozoa were immediately observed under a fluorescence microscope (Nikon Eclipse Ti, Tokyo, Japan) at a magnification of 600x using excitation/emission wavelengths of 488nm/530nm. The percentage of spermatozoa with normal/abnormal chromatin structure was determined by scoring a minimum of 100 spermatozoa/sample with bright, green fluorescence (double-stranded DNA) and those with orange/yellow fluorescence (single-stranded DNA).

### 2.12 Evaluation of protamine deficiency by chromomycin A3 staining

Air-dried and washed ZP-bound and unbound spermatozoa were fixed with Carnoy’s solution (acetic acid-methanol in a 3:1 ratio) at 37°C for 10 min. The fixed spermatozoa were incubated with 0.25mg/ml CMA_3_ (Sigma) in Mcllvaine buffer 0.1M citric acid and Na_2_HPO_4_•7H_2_O, pH 7.0, containing 10 mM MgCl_2_ for 20 min at room temperature. The slides were gently rinsed with PBS and mounted with fluorescent mounting medium (Dako). The stained spermatozoa were observed under a fluorescence microscope (Nikon Eclipse Ti, Tokyo, Japan) at a magnification of 600x using excitation/emission wavelengths of 488nm/580nm. The degree of protamine deficiency was determined by quantifying the overall signal intensity of a minimum of 100 spermatozoa with yellow fluorescence (CMA3-positive).

### 2.13 Evaluation of methylation level by immunostaining

Air-dried and washed ZP-bound and unbound spermatozoa were fixed with Carnoy’s solution (acetic acid- methanol in a 3:1 ratio) at 4°C for 20 min and gently rinsed with PBS with 0.5% Tween twice. For sperm decondensation, the fixed spermatozoa were incubated with 1M Tris-HCl, pH 9.5, containing 25 mM dithiothreitol (DTT) for 20 min at room temperature and gently rinsed with PBS twice. The decondensed spermatozoa are denatured by incubation with 6N HCl for 15 min and gently rinsed with PBS twice. The denatured spermatozoa were stained with anti-5-methylcytosine (5-mC) (Abcam) overnight at 4°C and gently rinsed with PBS twice. The samples were then incubated with Alexa Fluro 488 conjugated goat anti-mouse IgG for one hour at 37°C (1:1000; Invitrogen), rinsed gently with distilled water twice and immediately mounted with fluorescent mounting medium (Dako). The stained spermatozoa were observed under a fluorescence microscope (Nikon Eclipse Ti, Tokyo, Japan) at a magnification of 600x using excitation/emission wavelengths of 488 nm/525 nm. The level of methylation in spermatozoa was determined by quantifying the overall signal intensity of a minimum of 100 spermatozoa with green fluorescence (5-Mc-positive).

### 2.14 Data analysis

All experimental data were expressed as mean ± standard deviation (Mean ± SD) or median (range). Statistical software (GraphPad Prism 9.1.0, GraphPad software, CA, USA) was used to analyze the data. Two tailed unpaired t-test was used to examine the differences between the ZP-bound and unbound sperm subpopulations. If the data failed the normality test, Mann-Whitney (non-parametric) test was used to perform statistical analysis. A probability value of < 0.05 was considered as statistically significant.

## 3 Results

### 3.1 Retrieval of ZP-bound human spermatozoa by continuous spermatozoa-ZP coincubations

**Figure 1:**
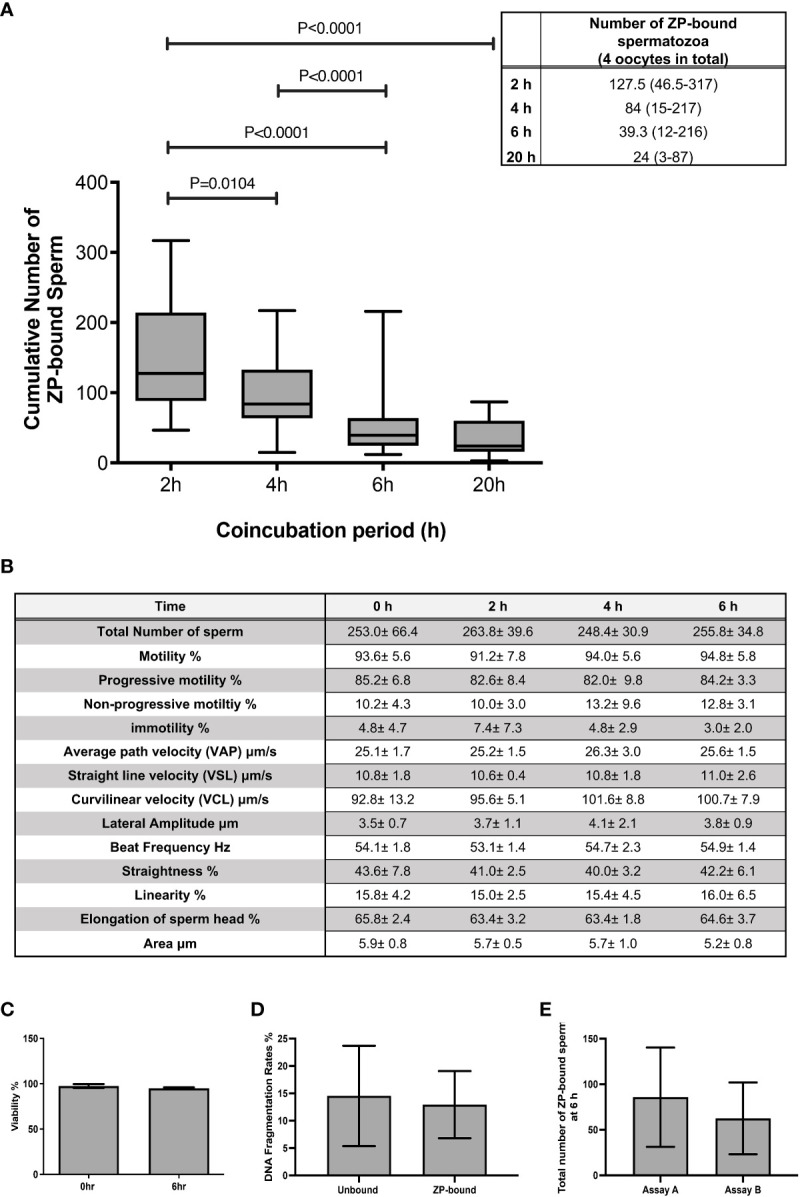
The total number of ZP-bound spermatozoa over the 6-h and 20-h incubation. **(A)** The number of spermatozoa tightly bound to the oocytes was counted at 2 h intervals during the 6-h incubation. There was a large variation in the number of ZP-bound spermatozoa, but the decreasing trend persisted in all samples. All data are represented as median (range) (n=24 for 6-h; n=6 for 20-h, p<0.05). **(B)** Effects of prolonged incubation on sperm motility at intervals of 0, 2, 4, 6 h. **(C, D)** Effects of prolonged incubation on sperm viability and DNA integrity at 0 and 6 hr. **(E)** The total number of ZP-bound spermatozoa with (Assay A) /without (Assay B) the replacement of fresh oocytes at 4 hr. All data are represented as mean ± SD (n=5).

The total number of spermatozoa tightly bound to the ZP per assay was quantified at 2 h intervals during the 6 h continuous coincubation (**Figure 1A**). An average of 150 ZP-bound spermatozoa was retrieved per assay within the first 2 h of coincubation. The time-course study showed that the total number of ZP-bound spermatozoa gradually decreased over time and eventually plateaued at 20 h (**Figure 1A**). Approximately 0.03% of the motile spermatozoa in the incubation droplet containing 1x10^6^ spermatozoa/mL bound to the ZP within the first 6-h incubation period. Incubation of spermatozoa for up to 6-h had no adverse effects on sperm viability, motility and DNA integrity (**Figures 1B-D**). The number of ZP-bound spermatozoa in two continuous assays with or without the replacement of oocyte at 4-h was comparable (**Figure 1E**), suggesting that reusing the same group of oocytes throughout the entire incubation period did not impair the binding interaction.

### 3.2 Immunodetection of acrosome reaction and protein markers on ZP-bound spermatozoa

**Figure 2:**
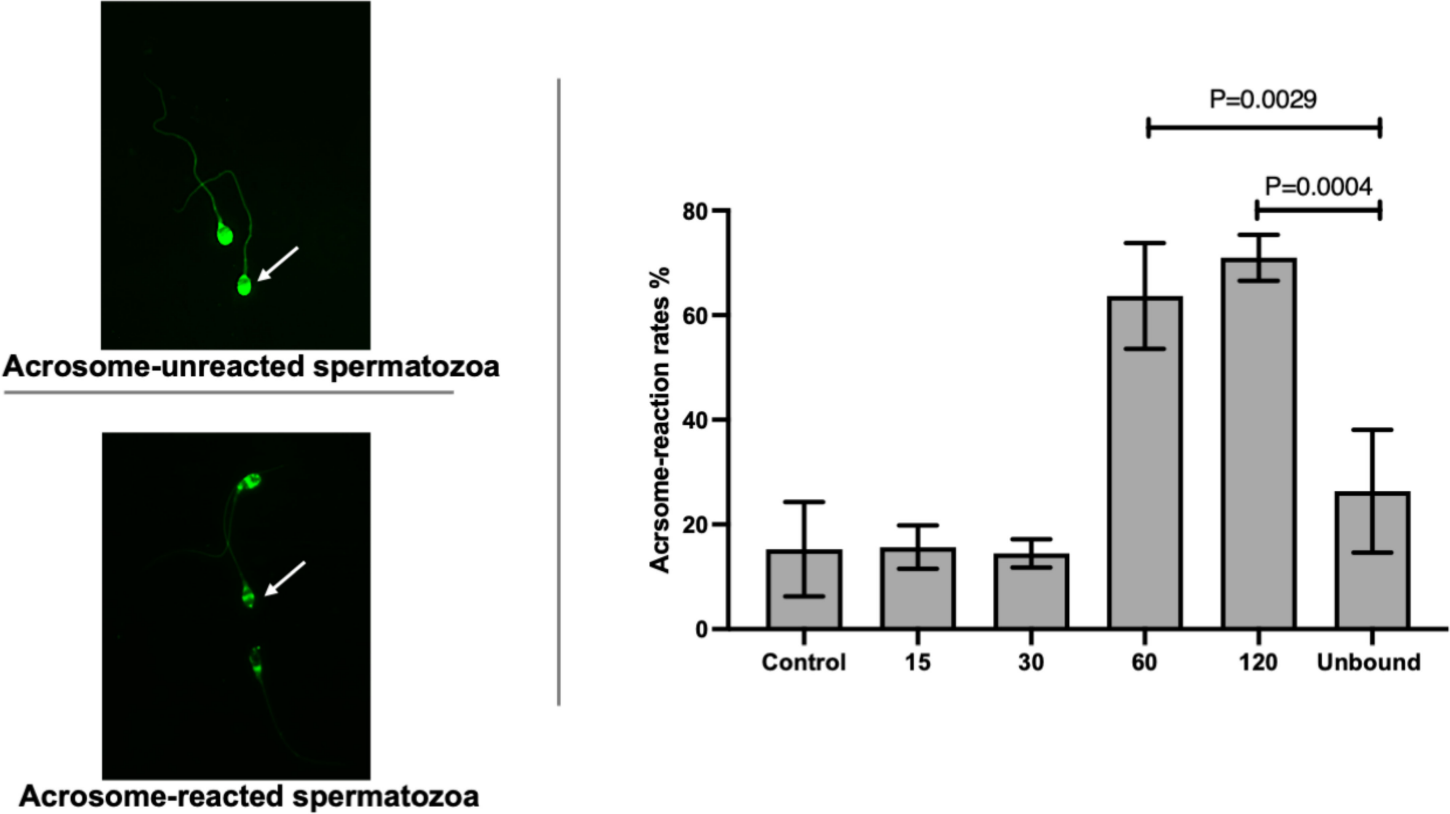
Time-course study of acrosome-reaction rates in ZP-bound spermatozoa. Acrosomal status of the control, unbound spermatozoa, ZP-bound spermatozoa (recovered at 15, 30, 60 and 120min) was determined by FITC-PSA. All data are represented as mean ± SD (n=4, p<0.05).

The acrosome reaction rates of ZP-bound spermatozoa recovered at 15 and 30 min (15.7% ± 4.2 and 14.5% ± 2.7) (**Figure 2**) were comparable to the spontaneous acrosome reaction rates of those in the control without prior exposure to the ZP. The ZP-bound spermatozoa recovered at 60 and 120 min (63.7% ± 10.1 and 70.9% ± 4.4 vs. 26.3% ± 11.7, p<0.05) (**Figure 2**) had higher acrosome reaction rates than the unbound ones.

**Figure 3:**
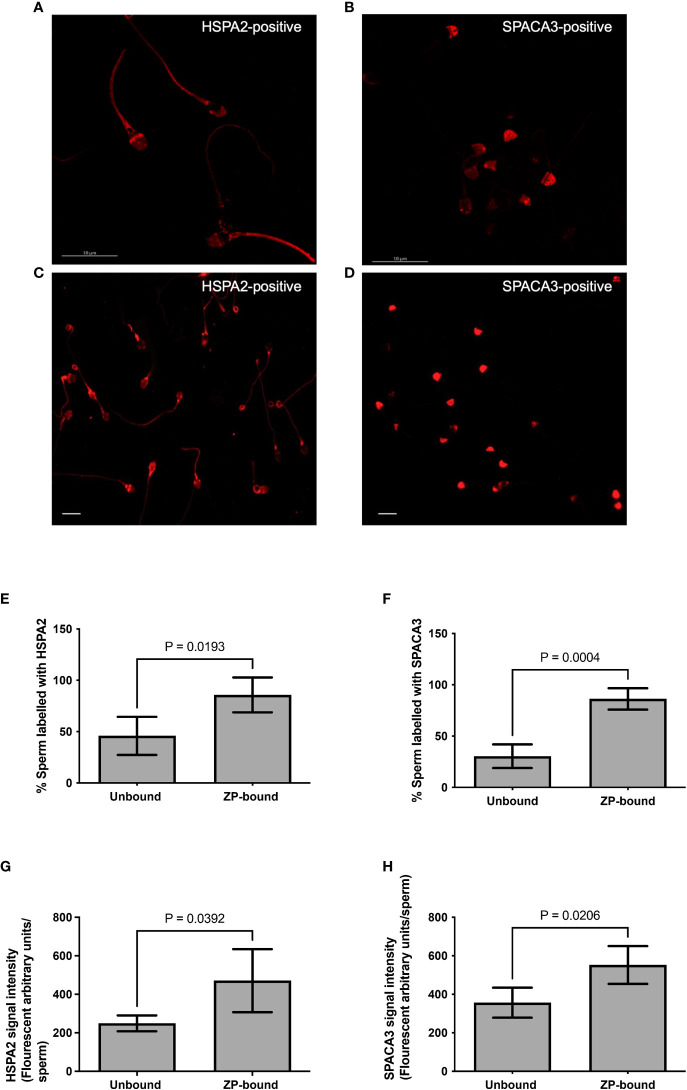
Immunolocalization of HSPA2 and SPACA3 on capacitated human spermatozoa. **(A, B)** Capacitated spermatozoa were labelled with anti-HSPA2 and SPACA3 antibodies (diluted 1:100) respectively followed by Alexa-555 secondary antibodies. The images captured using confocal microscopy showed granular staining patterns of HSPA2 and SPACA3 on spermatozoa. Scale bar = 10 μM. **(C)** HSPA2 was localized to the peri-acrosomal region, equatorial segment, post-acrosomal region, mid-piece and the sperm tail. **(D)** SPACA3 was localized to the acrosomal region. Scale bar=200 uM. **(E, F**) Quantification of ZP-bound recovered at 30 min and unbound spermatozoa showing positive staining with HSPA2 and SPACA3 at the head regions. All data are represented as mean ± SD (n=4, p<0.05). **(G, H)** Fluorescence intensity analysis of each protein marker was performed by the Image J software. All data are represented as mean ± SD (n=4, p<0.05).

The expression of HSPA2 and SPACA3 on capacitated human spermatozoa were individually evaluated by flow cytometry. The protein markers were positively present (HSPA2: 36.3 ± 6.5% and SPACA3: 17.9 ± 0.5%) (**Supplementary Figure 1**) on the capacitated human spermatozoa. Confocal microscopy revealed markedly granular pattern of staining for these molecules on capacitated spermatozoa (**Figures 3A, B**). HSPA2 and SPACA3 localized to the head regions of capacitated human spermatozoa (**Figures 3C, D**). To examine their expression solely on the plasma membrane, the optimal time-point to recover acrosome-intact, ZP-bound spermatozoa was 30 min. Approximately 86% of the ZP-bound spermatozoa recovered at 30 min were HSPA2- or SPACA3- positive (**Figures 3E, F**). ZP-bound spermatozoa recovered at 30 min had a higher percentage of positive HSPA2 (85.8% vs. 45.9%, p<0.05) and SPACA3 signals (86.3% vs. 30.5%, p<0.05) than the unbound ones (**Figure 3**). The signal intensities of both markers were stronger in the ZP-bound spermatozoa recovered at 30 min than the unbound ones (p<0.05) (**Figures 3G, H** **).**

### 3.3 Morphology evaluation of ZP-bound and unbound spermatozoa

**Figure 4:**
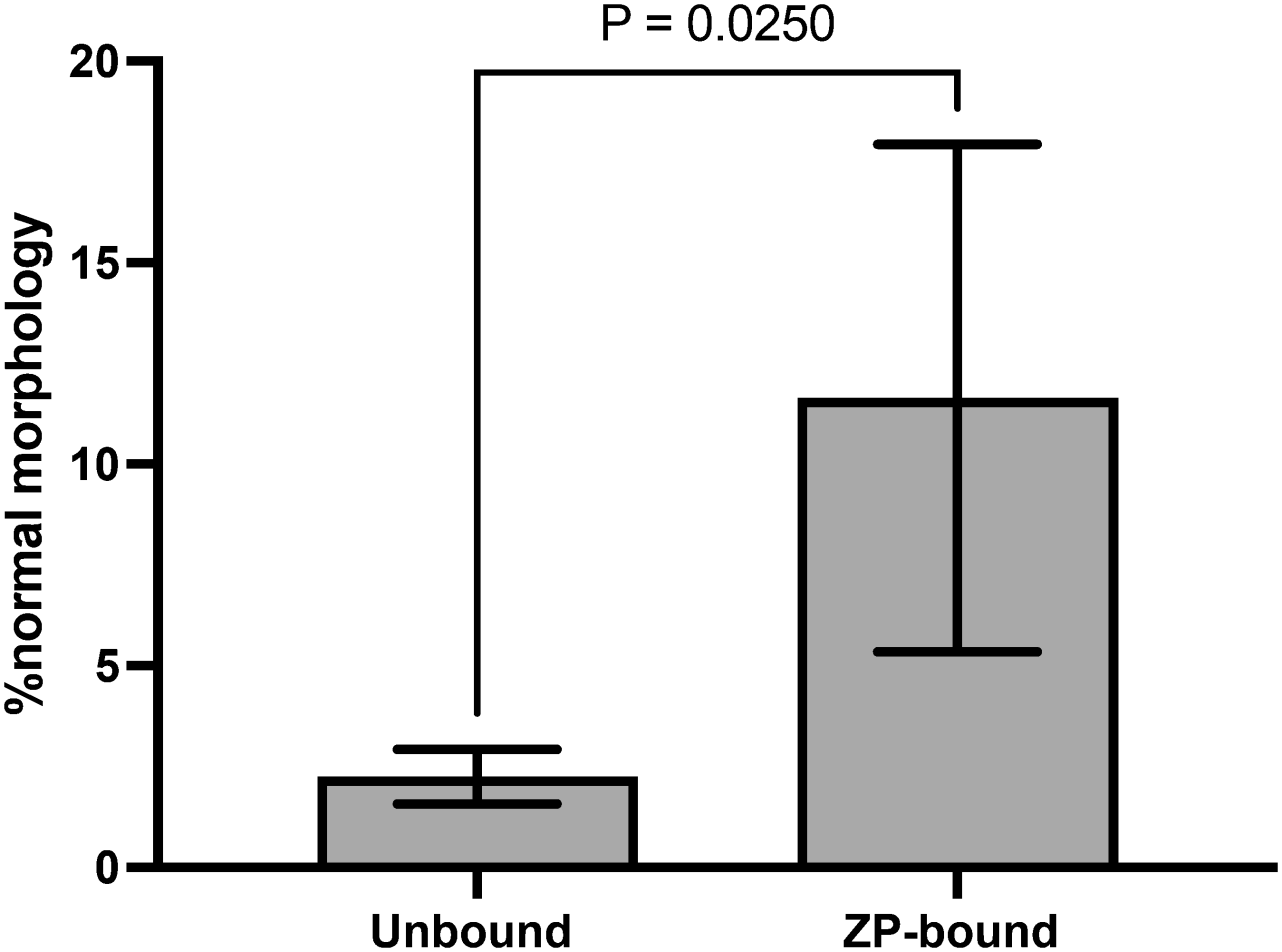
Morphology evaluation of ZP-bound and unbound spermatozoa. ZP-bound and unbound spermatozoa were classified as having normal or abnormal morphology according to the WHO strict criteria. All data are represented as mean ± SD (n=5, p<0.05).

The number of morphologically normal spermatozoa was significantly higher in the ZP-bound spermatozoa recovered at 30 min than in the unbound ones (**Figure 4**), indicating a relationship between the morphology and ZP-binding ability of spermatozoa.

### 3.4 Evaluation of genetic quality of ZP-bound and unbound spermatozoa

**Figure 5:**
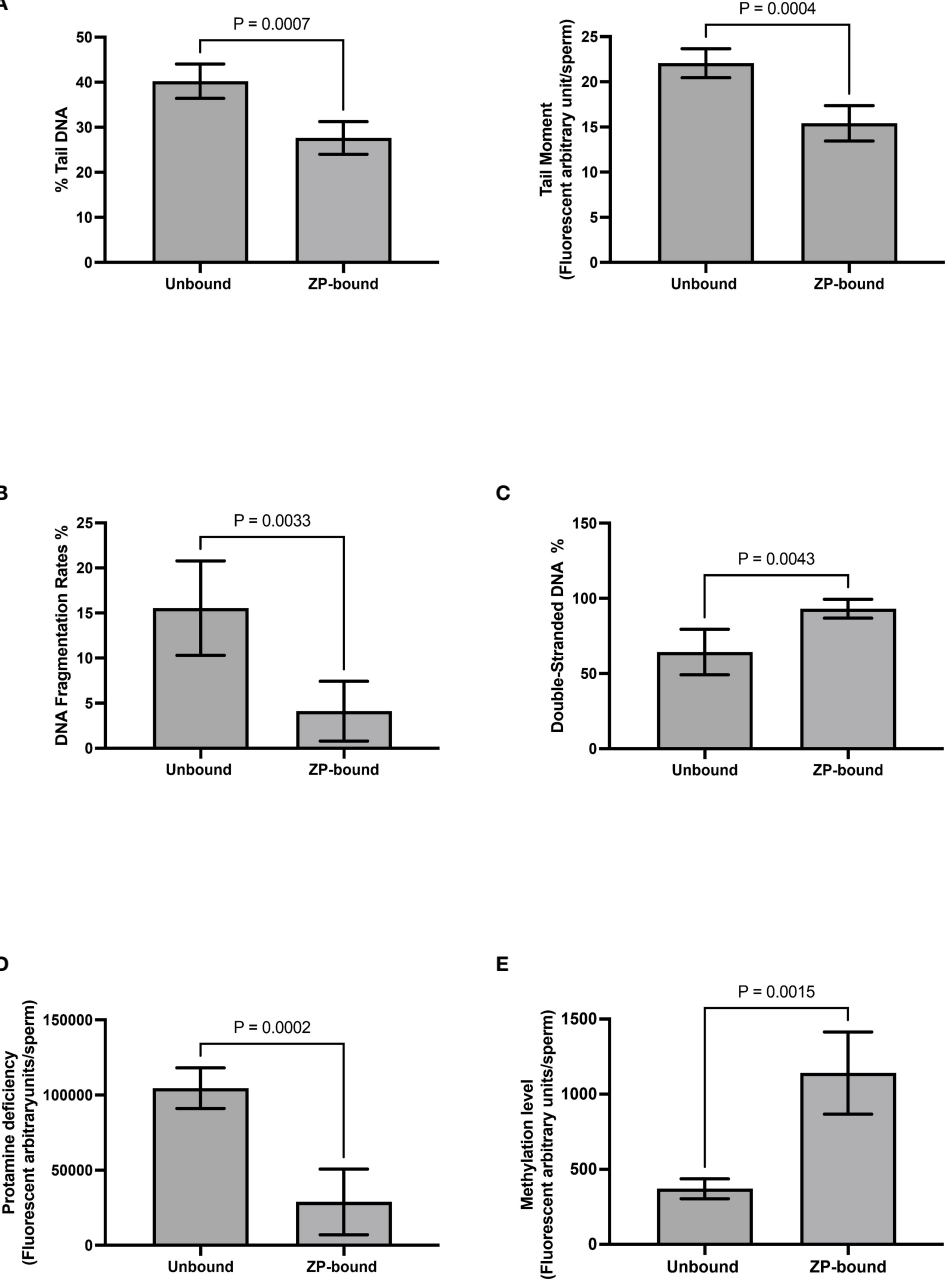
Evaluation of genetic qualities and epigenetic profile of ZP-bound and unbound spermatozoa. Quantification of **(A)** Comet-positive spermatozoa with different levels of DNA damages as reflected by the (Left) %tail DNA and (Right) tail moment **(B)** TUNEL-positive spermatozoa **(C)** AO-stained spermatozoa with double-stranded DNA in green fluorescence **D)** CMA3-positive spermatozoa with different levels of protamine deficiency as reflected by the yellow fluorescence intensity. **(E)** 5-Mc-positive spermatozoa with different levels of methylation as reflected by the green fluorescence intensity. All data are represented as mean ± SD (n=5 for **(A–E)**, p<0.05).

Comet and TUNEL assays (**Supplementary Figures 2A, B**) were used to evaluate DNA fragmentation rates of ZP-bound and unbound spermatozoa. In the comet assay, the ZP-bound spermatozoa had a significantly lower level of tail DNA (27.6% vs. 40.2%, p<0.05) (**Figure 5A**) and tail moment (15.4% vs 22.1%, p<0.05) (**Figure 5A**) than the unbound ones. Consistently, the number of TUNEL-positive spermatozoa was significantly lower in the ZP-bound than in the unbound sperm subpopulations (4.1% vs. 15.5%, p<0.05) (**Figure 5B**), correlating the ZP-binding ability and DNA integrity of spermatozoa. A high proportion of ZP-bound spermatozoa with intact DNA was found in all sample groups, even in raw semen with a high DNA fragmentation rate of 19.1% (**Supplementary Figure 3**). AO staining (**Supplementary Figure 2C**) was used to evaluate the chromatin structure and to differentiate spermatozoa with normal double-stranded DNA from those with abnormal single-stranded DNA. The number of spermatozoa with green fluorescence was significantly higher in the ZP-bound than the unbound spermatozoa (93.1% vs. 64.3%, p<0.05) (**Figure 5C**). There was a small proportion of ZP-bound spermatozoa with red fluorescence (6.4% on average) in both swim-up and raw sample groups (**Supplementary Figure 3** **)**, indicating a correlation between the ZP-binding ability and chromatin integrity of spermatozoa.

### 3.5 Evaluation of protamination and methylation of ZP-bound and unbound spermatozoa

CMA3 staining (**Supplementary Figure 2D** **)**was used to indirectly evaluate the degree of protamination in spermatozoa. CMA3 is a fluorochrome that specifically binds to the same location as protamines at the GC-rich sequences. Spermatozoa with protamine deficiency appeared bright yellow whilst those with proper protamination fluoresced light yellow with different degrees of signal intensity (**Figure 5D** **)**. The level of CMA3 positivity was significantly lower in ZP-bound than in the unbound spermatozoa (p<0.05) (**Figure 5D** **)**, suggesting that the human ZP selectively bound to spermatozoa with high degree of protamination. Immunofluorescent staining for the methylated regions (**Supplementary figure 2E** **)** was used to evaluate the overall methylation level of ZP-bound and unbound spermatozoa. Spermatozoa displayed varying degrees of green fluorescence depending on the DNA methylation level (**Figure 5E**). The overall methylation level was higher in ZP-bound than in the unbound spermatozoa (**Figure 5E**), as reflected by the signal intensity.

## 4 Discussion

Spermatozoa-ZP binding assay was first established by Overstreet and Hembree to examine the interaction between the non-viable oocytes and spermatozoa *in vitro* (29). Considering the small number of motile spermatozoa with ZP-binding ability in fertile donors (25, 30), it is logical to assume that this species-specific interaction might shed light on the potential use of ZP to select high-quality spermatozoa *in vitro*. In this study, the ZP-bound spermatozoa were found with higher rates of normal morphology, acrosome reaction rates, and DNA integrity when compared with the controls, consistent with previous studies demonstrating the selective nature of ZP to spermatozoa to fertilization-competent spermatozoa (31–33). Clinical studies using ZP-selected spermatozoa demonstrated minimal advantages on the fertilization and implantation rates but greatly improved the embryo qualities and pregnancy rates (34–37).

In this study, a modified continuous coincubation assay was used to collect spermatozoa without ZP-binding ability by gradually removing the ZP-bound spermatozoa from the incubation droplet at 2-h intervals over a 6-h period. Our results demonstrated that the total number of ZP-bound spermatozoa varied distinctively among samples, consistent with previous findings reporting on the differences in the number of motile spermatozoa with ZP-binding ability between fertile and infertile men (25). The time-course data indicated that about 50% of the motile spermatozoa with ZP-binding ability bound to the ZP within the first 2 h and gradually decreased over time until the end of coincubation at 6 hr. In comparison with a previous study (25), the overall percentage of ZP-bound spermatozoa recovered by our protocol was significantly lower (0.03% vs. 14.0% in fertile men). The discrepancy is likely due to the difference in protein concentration of BSA or other specific molecules related to ZP-binding ability. The number of ZP-bound spermatozoa is higher in culture medium supplemented with human serum than with BSA (38). Liu et al. also reported that the number of ZP-bound spermatozoa exponentially increased over 6 h, which differed from our results. Capacitation is a time-dependent process (39). It is possible that the components of the culture medium could affect capacitation during which the spermatozoa acquire ZP-binding ability (40), although human spermatozoa tend to complete capacitation within 3 h (41, 42). We also performed additional tests to confirm that the repeated uses of oocytes did not affect the ZP-binding results and that the extended incubation had no negative impact on the sperm parameters, excluding the possibility that the low ZP-binding efficiency was caused by progressive loss of functional and/or structural properties of the ZP and spermatozoa during incubation.

Spermatozoa-ZP binding interaction involves the recognition of ligands by the protein receptors on the plasma membrane of capacitated spermatozoa. Ultrasensitive analysis by mass spectrometry revealed that human ZP glycans are terminated with a high abundance of Siayl-LewisX (SLeX) sequences (43). Treatment targeting against SLeX sequences inhibits approximately 70% of spermatozoa-ZP binding (43). Moreover, recombinant human ZP-proteins with glycosylation different from their native counterparts are capable of binding to human spermatozoa (44, 45). The results suggested that both glycan-protein and protein-protein interactions are involved in spermatozoa-ZP binding. Proteomic studies identified a list of potential ZP receptors located on the plasma membrane of capacitated spermatozoa (46–48). During capacitation, dynamic changes occur on the sperm proteome to facilitate conformational modifications or exposure of ZP receptors located on the plasma membrane of acrosome-intact spermatozoa (49, 50). Soon after ZP-binding, acrosome reaction is initiated in ZP-bound spermatozoa to facilitate the subsequent penetration through the ZP. Thus, the acrosomal status is an indirect evaluation of sperm fertilization potential. Many membrane proteins, such as sperm adhesion molecule 1 (also known as PH-20), are also found on the inner acrosomal region of spermatozoa, which become exposed after the ZP binding interaction (51). In this study, we determined the optimal time-point to collect acrosome-intact, ZP-bound spermatozoa. Acrosome reaction is a time-dependent process. Consistently, after binding to the ZP, mouse spermatozoa remain acrosome-unreacted for approximately 40 min before commencing acrosome-reaction (52). Using the time-lapse fluorescence microscopy, it has been demonstrated that calcium ionophore (A23187) and progesterone induce acrosome reaction in human spermatozoa within 12 min (53). There is a lack of time-lapse study on the acrosome reaction rates of ZP-bound spermatozoa in humans. Our results demonstrated that majority of spermatozoa bound to the ZP for less than 30 min were acrosome-intact and approximately 60% of the ZP-bound spermatozoa became acrosome-reacted after 60 min. The ZP-bound spermatozoa recovered at 60 and 120 min had significantly higher acrosome reaction rates than the unbound ones.

In this study, the ZP-bound spermatozoa were recovered within 30 min to maximize the possibility of collecting acrosome-intact spermatozoa. The expression levels of HSPA2 and SPACA3 were about two times higher on the ZP-bound spermatozoa than on the unbound ones. HSPA2 is a testis-enriched member of the 70 kDa heat shock proteins (HSP-70) family, which promotes proper protein folding, translocation of intracellular proteins, and assembly of multimeric protein complexes (54). In humans, the HSPA2 gene expression is downregulated in men with azoospermia (55), varicocele and oligozoospermia (56), and idiopathic oligoteratozoospermia (57). The expression level of HSPA2 is positively correlated with maturity (58, 59), ability of binding to ZP and cumulus complexes (58, 60), and fertilization potential (58, 61–63) of human spermatozoa. Emerging studies suggest that HSPA2 remains predominantly intracellular in human spermatozoa, which serves as a chaperone to mediate the assembly of multimeric protein complexes located on the anterior region of the sperm head for ZP binding (64–66). During capacitation, HSPA2 is translocated from the inner to the outer leaflet of the plasma membrane, leading to an increased level of HSPA2 on spermatozoa (62, 67). HSPA2 interacts with the sperm adhesion molecule 1 (SPAM1) and arylsulfatase A (ARSA) to form an acrosomal domain for spermatozoa-ZP recognition. A lack of HSPA2 is found in infertile patients with defective ZP-binding ability (64). In this study, our results suggested that the expression level of HSPA2 on spermatozoa was reflective of their ZP-binding ability.

SPACA3, also known as SLLP1, is highly homologous with c-lysozyme and retains the putative substrate-binding residues conserved across mammals, such as humans and mice, for the binding to oligosaccharides of N-acetylglucosamine (GlcNAc) (68, 69). In mice, SLLP1 specifically binds to the receptors within the perivitelline space and on the plasma membrane of oocyte *in vitro* (70). The recombinant SLLP1 and antibodies directed against SLLP1 suppress the binding of spermatozoa to cumulus intact and ZP-free oocytes (70). This observation implies a functional role of SLLP1 in spermatozoa-oolemma interaction during fertilization. It is logical to assume that SLLP1 can participate in the spermatozoa-ZP binding interaction since GlcNAc has been identified on mammalian ZP (71–73). Partial inhibition of spermatozoa-ZP binding occurs when capacitated spermatozoa are pre-treated with GlcNAc or ZP is pre-incubated with glycosidase N-acetylglucosaminidase *in vitro* prior to hemizona assay (74, 75). The involvement of SLLP1 in spermatozoa-ZP interaction was further supported by a modified spermatozoa-ZP binding assay in which the calcium ions in the culture and co-incubation medium were replaced by the strontium ions to ensure that the spermatozoa were allowed to undergo capacitation required for ZP- binding, but not acrosome reaction following the binding interaction (76). This experimental condition demonstrated the inhibitory effects of exogenous GlcNAc specifically on spermatozoa- ZP binding (76). According to our results, neither HSPA2 nor SPACA3 were uniformly expressed on the entire population of ZP-bound spermatozoa, corroborating previous findings that the absence of a single protein receptor is highly unlikely to cause complete failure of spermatozoa-ZP binding because the ZP-receptor is a multimolecular structure assembled during capacitation (23, 77). Nevertheless, the expression level of a single marker could reflect the ability of spermatozoa acquiring the appropriate molecules required for ZP-binding during transit through the reproductive tract. The fluorescence intensity of protein markers can serve as an additional surrogate marker relevant to sperm fertilizing capability and genetic quality, which might in turn increase the discriminating power of the conventional semen analysis. If the protein markers are to become useful as diagnostic tools, clinical samples with varying degrees of ZP-binding ability should be collected to establish clinical thresholds of protein expressions.

DNA damages in spermatozoa are primarily induced by abortive apoptosis during spermatogenesis, chromatin remodeling during spermiogenesis, and oxidative stress during migration through the male reproductive tract (78). Under physiological circumstances, a low level of reactive oxygen species (ROS) is needed for physiological functions such as cellular activities and signaling pathways in spermatozoa (79). Oxidative stress as a result of excessive ROS production is closely related to male infertility (80). In some cases, spermatozoa with genetic abnormalities can retain their fertilizing ability leading to fertilization success, but the genetic defects might later manifest themselves as late paternal effects (81), resulting in a higher rate of suboptimal embryo development, pregnancy failure and pregnancy complications (82, 83). The level of DNA fragmentation is associated with lower pregnancy rates in IVF cycles following conventional insemination but not in intracytoplasmic sperm injection cycles (84), although increased miscarriage rates (84) and reduced live birth rates (85) are observed in both conventional insemination and intracytoplasmic sperm injection cycles. The ZP-binding ability of spermatozoa was closely related to their DNA integrity and chromatin status. Both alkaline Comet and TUNEL assays are developed to examine the overall level of DNA damages in spermatozoa. Alkaline Comet assay is a relatively sensitive method for measurement of single and double-strand breaks in spermatozoa. In this method, the fragmented DNA migrate out of the sperm head towards the anode forming a comet tail under an applied electrical field. The degree of DNA fragmentation is reflected by the fluorescence intensity and the length of the Comet tail. A high degree of DNA fragmentation measured by the comet assay is correlated with the lower rates of fertilization, good embryo development and implantation (86). TUNEL assay involves the attachment of fluorescently modified nucleotide to the free 3’- OH terminal of single- and double-stranded DNA mediated by TdT (87). The DNA fragmentation index is negatively correlated with inferior sperm parameters in terms of concentration, viability, motility, and morphology (88, 89). In this study, both methods demonstrated that the number of spermatozoa with fragmented DNA was significantly lower in ZP-bound spermatozoa than the unbound ones, implicating the relationship between the ZP-binding ability and genetic quality of spermatozoa.

AO intercalates into normal double-stranded DNA as a monomer and fluoresces green as opposed to binding to the denatured, single-stranded DNA as an aggregate which fluoresces yellow to red depending on the extent of the damages (90). The number of spermatozoa with green fluorescence was significantly higher in the ZP-bound spermatozoa than in the unbound ones, suggesting that the spermatozoa-ZP interaction is highly selective for spermatozoa with normal, double-stranded DNA. In contrast to the TUNEL results, the AO staining results demonstrated that the swim-up method failed to eliminate spermatozoa with abnormal genetic integrity; the number of spermatozoa with yellow-red fluorescence in post-swim-up samples was comparable to those in raw samples, consistent with previous findings that showed the lack of correlation between motility and chromatin integrity of spermatozoa (91).

In addition to genetic integrity, the epigenetic patterns of spermatozoa have been linked to embryonic development, implantation success, and the offspring health following ICSI (92, 93). Spermatozoa undergo epigenetic modifications including chromatin remodeling, DNA methylation, and non-coding RNAs to regulate transcriptional activities and gene expression at post-transcriptional level (94, 95). Aberrant epigenetic profiles in spermatozoa have been implicated in male idiopathic infertility. Protamine protein 1 and 2 (P1 and 2) are equally distributed in spermatozoa of healthy men. P1/2 imbalance is associated with poor sperm parameters and male infertility (96–99). Furthermore, abnormal methylation patterns at specific loci, such as H-19 and mesoderm-specific transcript (MEST), have been found in men with male-factor infertility (100–103). Aberrant global methylation levels are associated with suboptimal sperm parameters (104–106) and male infertility (107, 108). Our results indicated that the ZP-binding ability of spermatozoa was closely related to their epigenetic patterns in terms of protamination degree and global methylation level. The examination of methylation status at specific loci might definitively answer the question as to whether hypomethylation or hypermethylation is the major factor leading to suboptimal ZP-binding ability. To pinpoint the specific locus associated with ZP-binding ability, future investigation is needed to examine the DNA methylation profile of ZP-bound spermatozoa by single-cell bisulfite sequencing (109, 110). While the complicated staining and quantification procedures restrict wide application of genetic evaluation in clinical settings, future investigation should seek to develop a highly robust, indirect evaluation of sperm quality metrics by image analysis of sperm morphology associated with genetic integrity (111) and epigenetic pattern to establish a comprehensive profile of clinical samples.

## 5 Conclusion

Although conventional semen analysis is useful for establishing a fertility profile of men, it fails to provide diagnostic information regarding the sperm fertilization potential. The spermatozoa-ZP interaction serves as an integral part of the natural sperm selection mechanisms *in vivo*, which can potentially be used for sperm evaluation and selection in clinical settings. An early detection of defective ZP-binding ability in men with normal semen parameters could be offered intracytoplasmic sperm injection instead of conventional insemination, preventing them from financial and psychological sufferings caused by the low or no fertilization rates following conventional insemination. HPSPA2 and SPACA3 are the protein markers associated with the ZP-binding ability of spermatozoa. There was a quantitative difference in the expression level of HSPA2 and SPACA3 between acrosome intact, ZP-bound and unbound spermatozoa. Although the ZP-receptor is likely a multi-molecular structure, the expression level of a single marker could reflect the ability of spermatozoa acquiring the appropriate molecules required for ZP-binding during transit through the reproductive tract. Our results demonstrated that the ZP-binding ability of spermatozoa was closely related to their genetic quality in terms of DNA integrity, chromatin structure, protamination degree and global methylation level. Human ZP was highly selective for genetically normal spermatozoa with distinct epigenetic profiles. If the spermatozoa-ZP interaction is to become useful as a clinical tool, clinical samples with varying degrees of ZP-binding ability should be collected to establish a representative database for identification of high-quality spermatozoa by image analysis of sperm morphology. Taken together, the development of a robust and reproducible selection method incorporating the ZP-binding ability of spermatozoa might improve the overall workflow and the pregnancy outcomes in ART.

## Data availability statement

The original contributions presented in the study are included in the article/**Supplementary Material**. Further inquiries can be directed to the corresponding authors.

## Ethics statement

The studies involving human participants were reviewed and approved by Institutional Review Board of the University of Hong Kong/Hospital Authority Hong Kong West Cluster. The patients/participants provided their written informed consent to participate in this study.

## Author contributions

WY and PC conceived and designed the project. EL, BL, and KL collected samples and conducted experiments. EL and BL analyzed and interpreted the collected data. EL and PC wrote the first draft of the manuscript. C-LL, XT, KL, RL, EN, WY, and J-PO revised the manuscript. All authors contributed to the article and approved the submitted version.
