# Supplementary material for: The role of spermatozoa-zona pellucida interaction in selecting fertilization-competent spermatozoa in humans
Source: Front Endocrinol (Lausanne). 2023 Mar 20;14:1135973. doi: 10.3389/fendo.2023.1135973 (PMC10067631; doi:10.3389/fendo.2023.1135973)
Supplement: Supplementary file 1 [file DataSheet_1.pdf]

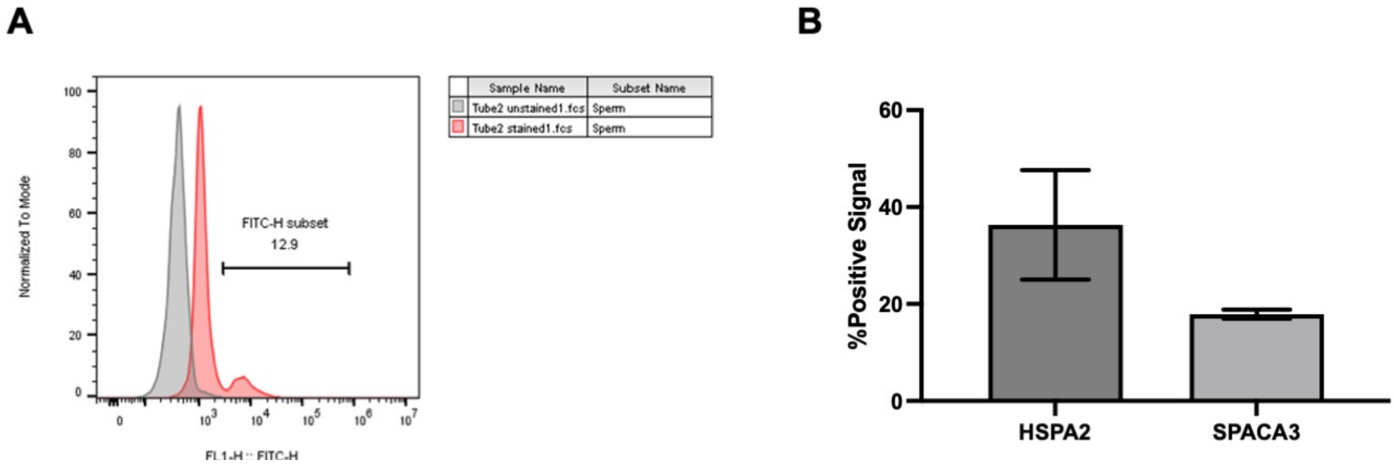

Supplementary figure 1. Evaluation of protein expressions on capacitated spermatozoa by flow cytometry. (A) Representative flow cytometry data from spermatozoa labelled with anti-HSPA2 or SPACA3 antibodies respectively (diluted 1:100) followed by Alexa-488 secondary antibodies. **(B)** HSPA2 and SPACA3 were positively detected on capacitated spermatozoa. All data are represented as mean ± SD (n=3).

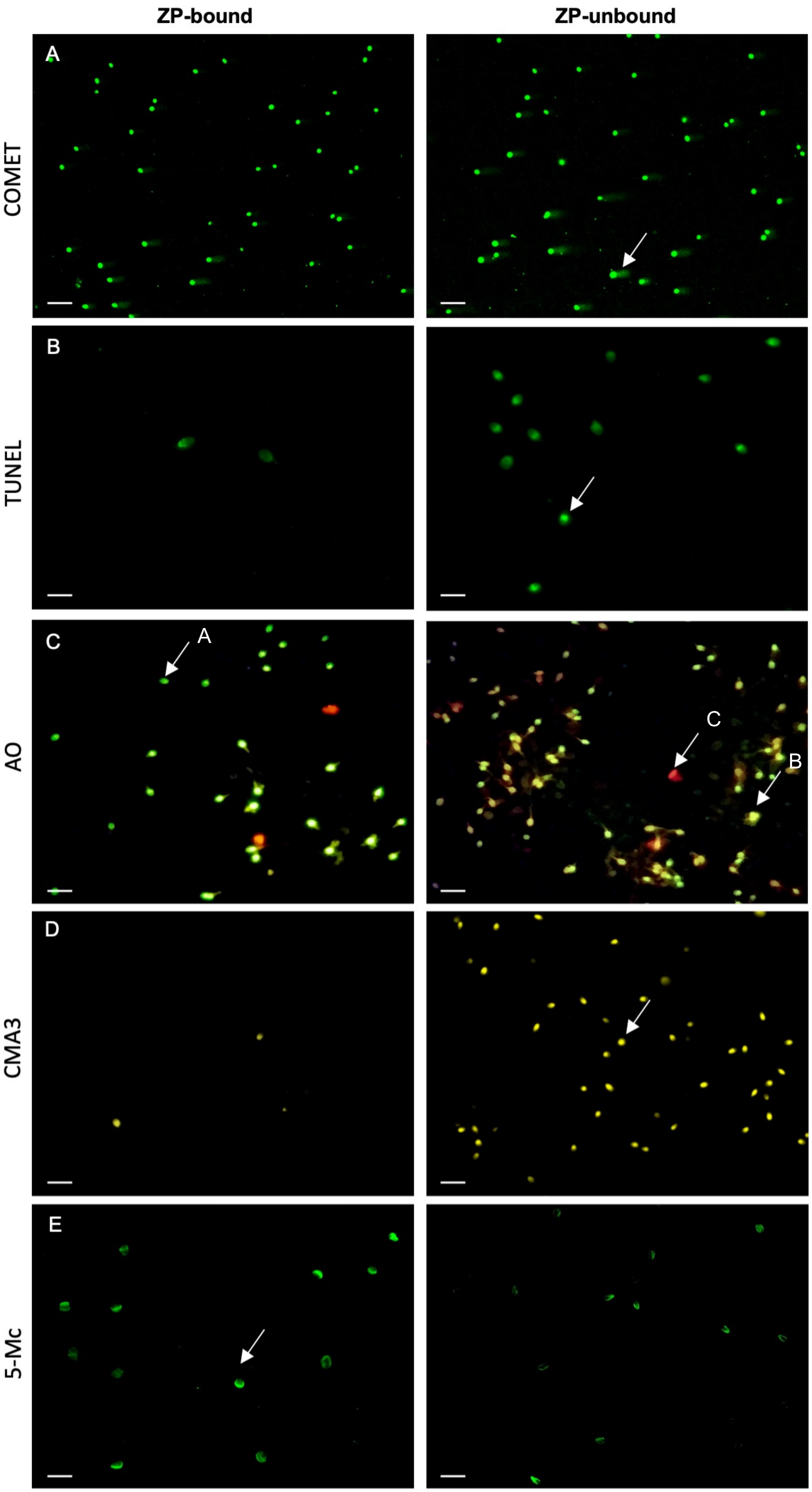

Supplementary figure 2. Representative images of **(A)** Comet-positive spermatozoa (arrow). (**B**) TUNEL-positive spermatozoa with bright, green fluorescence (arrow). **(C)** AO-stained spermatozoa with double-stranded DNA in green fluorescence (arrow A) and those with single-stranded DNA in yellow (arrow B) to red (arrow C) fluorescence depending on the extent of the damages.**(D)** CMA3-positive spermatozoa in yellow fluorescence (arrow). **(E)** 5-Mc-positive spermatozoa in bright, green fluorescence (arrow). Scale bar = 200 μM.

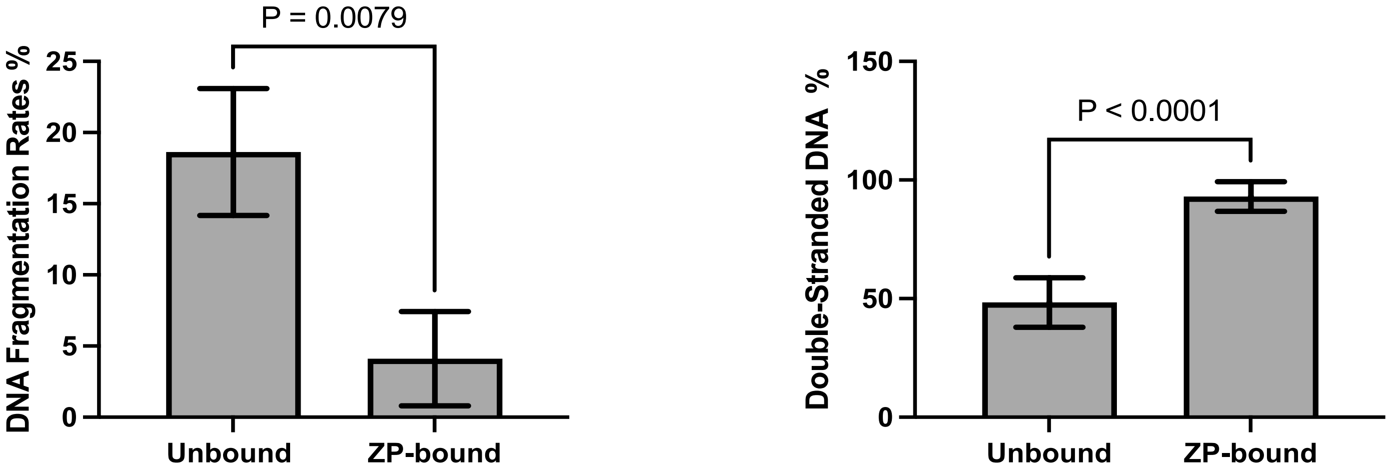

Supplementary figure 3. Evaluation of DNA integrity and chromatin structure of ZP-bound and –unbound spermatozoa recovered from raw sample. (A) DNA fragmentation rates of sperm subpopulations evaluated by TUNEL. (B) Percentage of double-stranded DNA of sperm subpopulations by AO. All data are represented as mean ± SD (n=4).
